# Validation of the Nelwan Score as a screening tool for the diagnosis of typhoid fever in adults in Indonesia

**DOI:** 10.1371/journal.pone.0256508

**Published:** 2023-05-12

**Authors:** Erni Juwita Nelwan, Luh Putu Listya Paramita, Robert Sinto, Decy Subekti, Fransiscus Nikodemus Hosea, Pringgodigdo Nugroho, Herdiman T. Pohan

**Affiliations:** 1 Division of Tropical and Infectious Disease, Department of Internal Medicine, Cipto Mangunkusumo Hospital, Jakarta, Indonesia; 2 Faculty of Medicine Universitas Indonesia, Jakarta, Indonesia; 3 Department of Internal Medicine, Cipto Mangunkusumo Hospital, Universitas Indonesia, Jakarta, Indonesia; 4 Oxford University Clinical Research Unit, Faculty of Medicine Universitas Indonesia, Jakarta, Indonesia; 5 Division of Nephrology and Hypertension, Department of Internal Medicine, Cipto Mangunkusumo Hospital, Jakarta, Indonesia; Universitas Padjadjaran, INDONESIA

## Abstract

**Introduction:**

Typhoid fever diagnosis is challenging for clinicians in areas with limited laboratory facilities. Scoring methods based on signs and symptoms are useful for screening for probable cases of typhoid fever. The Nelwan Score variables are derived from the clinical signs and symptoms of patients with suspected typhoid. We validated the Nelwan Score compared to laboratory tests as the gold standard.

**Methods:**

This cross-sectional study was conducted between July 2017 and January 2018 in five hospitals and two primary health care centers in Jakarta and Tangerang, Indonesia. Patients with fever for 3–14 days and gastrointestinal symptoms were evaluated using the Nelwan Score. Blood cultures, samples for polymerase chain reaction testing, and additional rectal swab cultures were collected simultaneously to confirm the diagnosis of typhoid. Data were analyzed using a contingency table to measure sensitivity, specificity, positive predictive value (PPV), and negative predictive value (NPV), and the optimal cut-off of the Nelwan Score for typhoid diagnosis was determined using a receiver-operating characteristic curve.

**Result:**

Typhoid was confirmed in 11 of the 233 patients (4.7%) with suspected typhoid. Among laboratory-confirmed typhoid cases, the median Nelwan Score was 11 (range: 9–13) and the optimal cut-off value was 10, with an area under the curve of 71.3%, sensitivity of 81.8%, specificity of 60.8%, PPV of 9.3%, and NPV of 98.5%.

**Conclusion:**

A Nelwan Score of 10 is the best cut-off value for screening for typhoid fever. It is useful as screening tool for typhoid fever, where laboratory resources are limited, and could help to decrease irrational antibiotic use.

## Introduction

Typhoid fever, caused by *Salmonella typhi* and *Salmonella paratyphi*, is an infectious disease with a heavy public health burden. Globally, 14.3 million cases were reported in 2017, with a case fatality rate of 0.95% (95% Uncertainty Interval [UI] (0.54–1.53%)) [1]. Typhoid fever is endemic in Indonesia and needs active attention because of the increasing annual incidence (500/100.000 population) and mortality rate of 0.6–5% [2]. In addition, screening for typhoid fever in 2013 showed that 2.9% of food vendors in Jakarta were typhoid carriers [2, 3].

The nonspecific clinical presentation of typhoid fever adds to the diagnostic difficulties because it mimics other febrile illnesses such as malaria, dengue fever, and influenza [4–7]. Nonetheless, characteristic presentations, such as “step-ladder” fever, relative bradycardia, and coated tongue, aid in clinical diagnosis. These features were described by Haq et al. [5] to have good specificity (100%, 94%, and 94% for “step-ladder” fever, relative bradycardia, and coated tongue, respectively). However, fever and abdominal pain were the most reported symptoms [8, 9]. In resource-limited settings, diagnosis is highly dependent on the clinical presentation. Therefore, careful evaluation is needed to distinguish typhoid fever from other diseases.

Laboratory investigations are important for screening and diagnosis of typhoid fever. However, serological tests, culture, and polymerase chain reaction (PCR) have diagnostic limitations, and investigations such as blood and bone marrow culture are not readily available in most typhoid-endemic countries. Likewise, the Widal test, which is more commonly used in endemic countries, has poor specificity and titer cut-off limitations. Another drawback is that laboratory investigations are costly and not always available, and this contributes to delayed diagnosis and inappropriate treatment [3, 8, 10, 11]. To overcome these diagnostic difficulties, several rapid diagnostic tests have recently been evaluated [12, 13]. However, their acceptance and widespread use in laboratories in typhoid-endemic developing countries are limited.

In Indonesia, antibiotics are prescribed for patients with clinically suspected typhoid because adequate laboratory diagnosis is lacking in most rural health facilities. Symptom-based treatment with antibiotics leads to an increased rate of antimicrobial resistance [14]. The WHO listing of *Salmonella* spp. as a high-priority pathogen facing antibiotic resistance is evidence of the administration of treatment on a clinical basis [15]. Despite efforts to support different methods of diagnosing and managing typhoid fever, the incidence, morbidity, and mortality rates remain high [2, 3, 9, 16]. Thus, prompt, accurate diagnosis is needed to decrease the typhoid fever burden, including a decrease in costly laboratory investigations and antimicrobial resistance.

The Nelwan Score, which was first developed by Nelwan in 1991 is a clinical scoring system that aids in the diagnosis of typhoid fever [17]. With this method, points are assigned to the signs and symptoms obtained from the history and physical examination. This scoring method has been used as a research tool in the diagnosis of typhoid fever in Indonesia and has not yet been applied in clinical case diagnosis in health facilities.

With the Nelwan Score, the points obtained are totaled, and the score corresponds to the probability of a clinical diagnosis of typhoid fever: a score of 13 or more is rated highly likely to be typhoid fever, and typhoid fever is unlikely if the score is < 7. Although the Nelwan Score can help in the diagnosis of typhoid fever, this scoring method has not been validated compared with blood culture and PCR as the reference standard for typhoid diagnosis. In this study, we validated the Nelwan Score for the diagnosis of typhoid fever in adults compared with laboratory tests.

## Methods

### Study design

A cross-sectional study was conducted between July 2017 and January 2018 using primary data of patients who visited the emergency department, outpatient clinic, and internal medicine ward in five hospitals (Persahabatan Hospital, Budhi Asih Hospital, South Tangerang Hospital, Hermina Ciputat Hospital, and Metropolitan Medical Center Hospital) and two primary health care centers (Jatinegara Primary Health Center and Gambir Primary Health Center) in the Jakarta area. The inclusion criteria were adult patients (aged 18–65 years) with fever ≥ 37.5°C, for 3 to 14 days, and at least one abdominal manifestation indicative of typhoid fever. Pregnant women, those with known causes of fever, and those treated with antibiotics were excluded.

Clinical history and physical examination were performed on all study participants with routine laboratory investigations (complete blood count and differential count). In addition, blood culture and PCR were carried out, and rectal culture was performed in participants who had fever for more than 7 days.

All participants were interviewed and the Nelwan Score was calculated (Table 1). Patients with fever and abdominal symptoms were considered clinically probable cases of typhoid fever, and the clinical diagnosis was confirmed by positive blood culture, rectal swab culture, or PCR of a blood sample.

**Table 1:** Symptoms and signs of the Nelwan Score for typhoid fever.

	Score
Symptom	
Fever ≤ 1 week	1
Fever >1 week	2
Headache	1
Weakness	1
Nausea	1
Abdominal pain	1
Anorexia	1
Vomiting	1
Motility Disorder	1
Insomnia	1
Sign	
Hepatomegaly	1
Splenomegaly	1
Relative bradycardia	2
Typhoid tongue	2
Melena stools	2
Impaired consciousness	2
Total score	20

Nelwan Score: The score was calculated by assigning a point to each of the following symptoms: fever for less than one week, headache, weakness, nausea, abdominal pain, anorexia, vomiting, disturbed gastrointestinal motility, insomnia, hepatomegaly, and splenomegaly (Table 1). Two points were assigned for fever for more than one week, relative bradycardia, typhoid tongue, melena stools, and impaired consciousness. The maximum possible score was 20. When first developed, a score of 13 or more indicated that a patient was highly likely to be positive for typhoid fever, a score ranging between 8 and 12 provided a 50% probability of typhoid fever positivity, and a score of 7 or less indicated that typhoid fever was unlikely.

### Laboratory investigations

**Blood culture.** An 8–10 mL sample of blood was collected using a universal standard antiseptic procedure and injected into BACTEC Plus Aerobic bottles, which were transported daily to the Eijkman Oxford Clinical Research Unit laboratory and processed according to standard procedures for the isolation and identification of *S*. *typhi* [18, 19]. The inoculated bottles were incubated in a BD BACTEC 9050 system instrument at 37°C for 7 days, or until they were detected as positive by the instrument. When bacterial growth was detected, small aliquots of medium were subcultured on MacConkey and *Salmonella*-*Shigella* (SS) agar plates. MacConkey and SS agar plates were incubated at 37°C for 18 to 24 hours. On MacConkey agar, *S*. *typhi* was identified as smooth, non-lactose-fermenting colonies. On SS agar, *S*. *typhi* was identified as a non-lactose-producing, non-fermenting colonies with a black center. Suspected colonies were screened using Kligler iron agar, motility indole ornithine, and citrate utilization tests. Colonies showing biochemical reactions suggestive of *S*. *typhi* were confirmed serologically by a slide agglutination test with Vi antiserum, *Salmonella* D1 group-specific antiserum, *Salmonella* O factor 9 antiserum (Becton Dickinson Laboratories, Franklin Lakes, NJ, USA).

#### Rectal swab culture

Rectal swabs were transported to the laboratory in Cary and Blair medium and plated directly on SS and MacConkey agar plates. Swab samples were also placed in *Salmonella*-selective enrichment broth (selenite cystine broth). After 18 to 20 h of incubation in the broth at 37°C, these swabs were plated on fresh sets of the same two agar media. Agar plates were also incubated at 37°C for 20 to 24 h. Identification of *S*. *typhi* was performed by selecting isolated colonies with characteristics of *S*. *typhi* as described in the previous section on blood culture examination [19].

#### DNA extraction and polymerase chain reaction

DNA was extracted from 200-μL blood samples using the QIAmp DNA mini kit (Qiagen, Hilden, Germany) according to the manufacturer’s instructions. PCR testing was performed using primer sets for flagellin genes of S. typhi: fliC and fliB (z66). The fliC gene was amplified with primers fliC_F: 5'-TTA-ACG-CAG-TAAAGA-GAG-3' and fliC_R: 5'-ATGGCA-CAA-GTC-ATT-AAT-AC-3', which produced 1521 base pairs for the d-allele and 1273 bp for the j-allele. Amplification of the fliB (z66) gene was performed with z66 flag_F: 5'-ATG-GCA-CAA-GTC-ATC-AAT-AC-3' and z66 flag_R: 5'-TTA-ACG-CAG-CAG-AGA-CAG-TAC3' produced 1479 bp amplicons [20, 21]. PCR was carried out with PCR toptaq DNA polymerase (Qiagen, Hilden, Germany) with a DNA concentration of 20 ng/mL, cycled 35 times on a Thermocycler PCR System 9700 (Applied Biosystems, Foster City, CA, USA), and the resulting products were analyzed on a 1% agarose gel with 2 μL loading dye (Promega, Madison, WI, USA), for each 5 μL sample. The size was measured by comparing the migration available with the 100 bp DNA ladder (Promega, Madison, WI, USA).

### Data analysis

Data analysis was performed using Stata version 15.0 (StataCorp, College Station, TX, USA). Descriptive analysis was performed on demographic data and clinical manifestations. The diagnostic value of the Nelwan Score was assessed by calculating the sensitivity, specificity, predictive value, and likelihood ratio, and cut-off analysis was performed by plotting the receiver-operating characteristic (ROC) curve with 95% confidence intervals (CI).

### Ethical considerations

Written informed consent was obtained from all participants prior to data collection. Ethical approval was obtained from the Ethical Board of Universitas Indonesia (approval number: 641/UN2). F1/ETIK/2017). All data collected were kept confidential.

## Results

A total of 233 participants were enrolled and assessed using both the Nelwan score and reference tests. The signs and symptoms reported by the participants are presented in Table 2. Of the 233 participants, 11 (4.7%) had typhoid confirmed by positive blood culture. However, none of the rectal cultures or blood PCR results were positive. Of the 11 participants with confirmed typhoid, six (55%) were female. The median age of all study participants was 38 years, whereas the median age of the patients with confirmed typhoid was 26 years.

**Table 2:** Clinical characteristics of study subjects (clinical and confirmed cases).

Clinical characteristics	Total	Typhoid confirmed	Typhoid not confirmed
	n = 233	n = 11	n = 222
Sex			
Male	110	5 (4.5%)	105 (95.5%)
Female	123	6 (4.88%)	117 (95.12%)
Age (years), median (range)		26 (20–45)	38 (18–81)
Fever ≤ 1 week	202 (86.7)	11 (100)	191 (86)
Fever > 1 week	31 (13.3)	0 (0)	31 (13.9)
Headache	209 (89.7)	11 (100)	198 (89.2)
Malaise	221 (94.8)	11 (100)	210 (94.6)
Nausea	222 (95.3)	11 (100)	211 (95)
Abdominal pain	207 (88.8)	11 (100)	196 (88.3)
Anorexia	211 (90.6)	11 (100)	200 (90.1)
Vomiting	168 (72.1)	10 (90.9)	160 (72.1)
Motility disorder	177 (76)	11 (100)	166 (74.8)
Insomnia	174 (74.7)	11 (100)	163 (73.4)
Hepatomegaly	7 (3)	0	7 (3.2)
Splenomegaly	0	0	0
Relative bradycardia	21 (9)	7 (63.6)	14 (6.3)
Typhoid tongue	94 (40.3)	10 (90.9)	84 (37.8)
Melena	21 (9)	0	21 (9.5)
Impaired consciousness	7 (3)	1 (9.1)	6 (2.7)
Nelwan Score, median (range)	9 (4–15)	11 (9–13)	9 (4–15)

Values are reported as n (%), unless indicated otherwise.

The symptoms of fever for less than a week, headache, weakness, nausea, abdominal pain, anorexia, motility disorder, and insomnia were present in all patients with laboratory-confirmed typhoid, thus, no participants with confirmed typhoid fever had a Nelwan Score less than 9.

**Fig 1:**
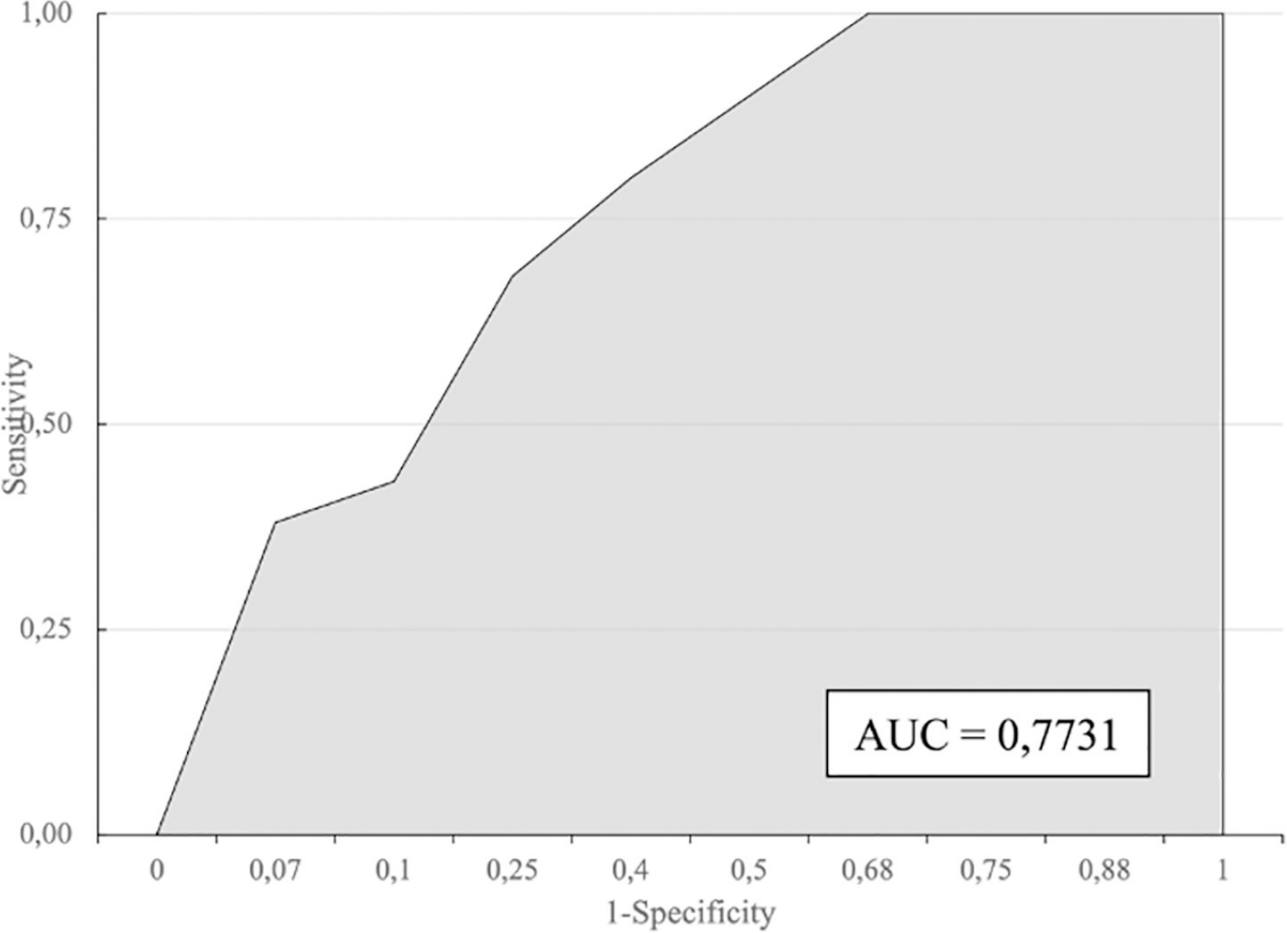
Receiving-operating characteristic (ROC) curve of the Nelwan Score for diagnosing typhoid fever.

Fig 1 shows the ROC curve analysis. The diagnostic prediction according to the area under the curve (AUC) was 77.3% (95% CI: 65.9–88.7%).

**Fig 2:**
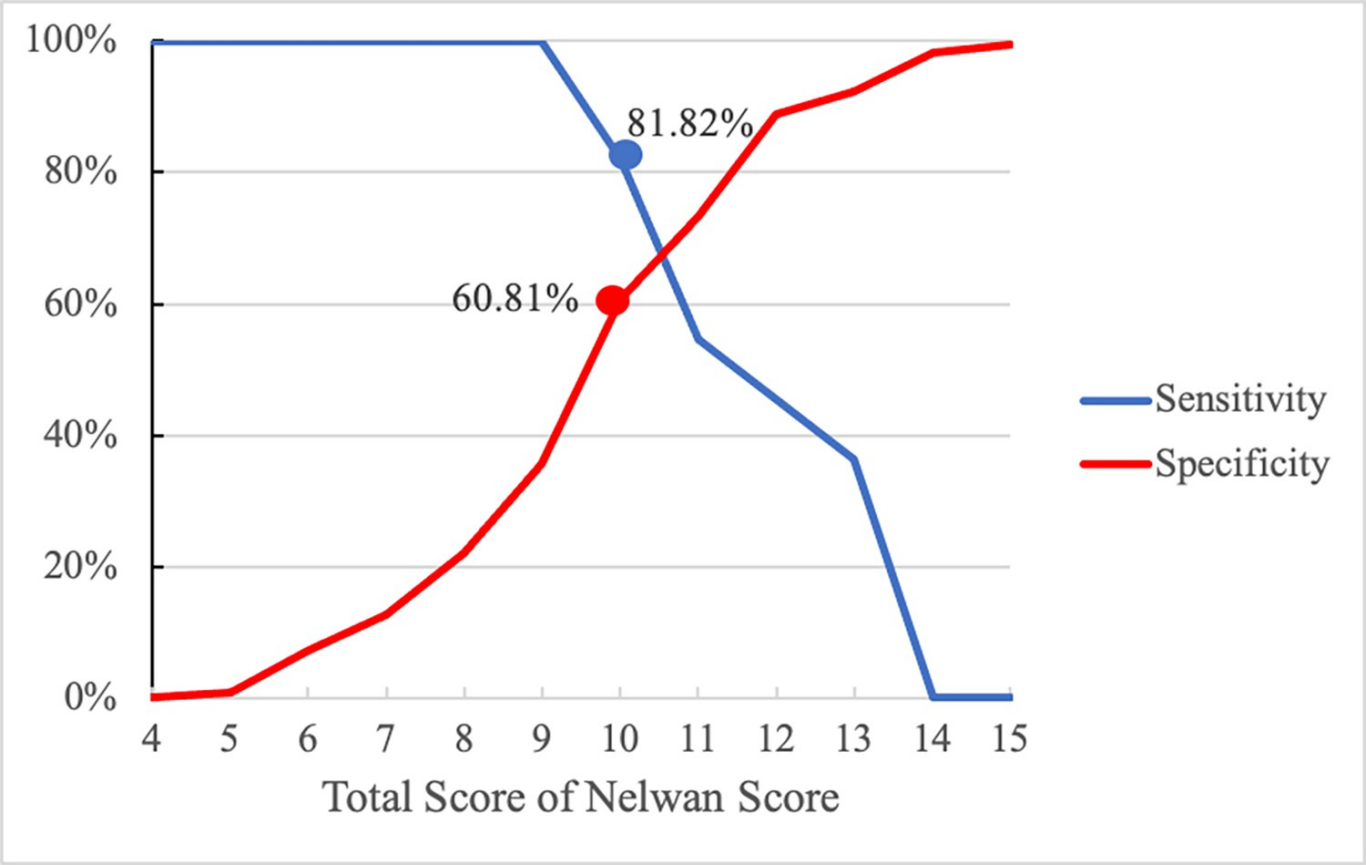
Receiver-operating characteristic curve of the Nelwan Score showing the intersection between sensitivity and specificity.

The corresponding cut-off values to estimate the typhoid fever diagnosis and risk in patients with fever are shown in Fig 2.

To explore the best diagnostic value of the Nelwan Score, we calculated different cut-off values. A value of 10 had the highest sensitivity (81.8*%)* with a negative predictive value (NPV) of 98.5% and reliability of 42.6%, whereas a sensitivity of 100% was obtained using at a cut-off value of 9, and specificity of 88.7% was observed using a cut-off value of 12 (Table 3).

**Table 3:** Diagnostic value of Nelwan Score.

Cut-off Value	Sensitivity % (95% CI)	Specificity % (95% CI)	PPV % (95% CI)	NPV % (95% CI)	LR+ (95% CI)	LR− (95% CI)	Reliability %
**10**	81.8 (52–95)	60.8 (54–67)	9.3 (5–17)	98.5 (95–100)	2.09 (1.66–2.62)	0.30 (0.19–0.47)	42.6
**11**	54.5 (28–79)	73.4 (67–79)	9.2 (4–19)	97 (93–99)	2.05 (1.47–2.87)	0.62 (0.47–0.82)	27.9
**12**	45.5 (21–72)	88.7 (84–92)	16.7 (7–34)	97 (94–99)	4.04 (2.33–7.00)	0.61 (0.44–0.86)	34.2

LR, likelihood ratio; NPV, negative predictive value; PPV, positive predictive value.

## Discussion

The Nelwan Score for the diagnosis of typhoid fever was evaluated based on the clinical signs and symptoms of patients who presented to five hospitals and two community health facilities in the greater Jakarta area. A Nelwan Score of 10 had an acceptable sensitivity (81.8%) and NPV (98.5%), and was the optimal cut-off value for diagnosing typhoid fever. These results suggests that the Nelwan Score has practical value as a screening tool that could benefit clinicians working in health facilities with limited laboratory facilities [17, 22]. A tool that aids in early diagnosis and prompt treatment of typhoid fever is essential in Indonesia to reduce morbidity and prevent transmission. As the treatment of typhoid fever includes the administration of antibiotics, making a reliable clinical diagnosis at presentation would prevent the irrational use of antibiotics that might contribute to antimicrobial resistance. Indonesia has already been identified as an epicenter for antimicrobial resistance [14].

The usefulness of the Nelwan Score was determined using an ROC curve. A cut-off value of 10 provided the best trade-off between sensitivity and specificity. As this is the first study to assess the optimal cut-off value of the Nelwan Score, a comparison could not be evaluated.

We compared our findings with those of several studies that focused on the clinical features of aiding typhoid fever diagnosis. Haq et al [5]. reported that clinical manifestations promoted a good diagnostic value for typhoid fever. However, their study reported the specificity of each separate clinical manifestation compared to microbiological results. Kuvandik et al. [6], reported that several relevant clinical manifestations might aid clinicians in diagnosing typhoid fever, combined with laboratory investigation. Kuvandik et al. [6] reported that clinical manifestations (splenomegaly, rose spots, and relative bradycardia) alone provided a high sensitivity (91.7%) but limited specificity (34.5%) for typhoid diagnosis in the absence of microbiological confirmation [6]. Hosoglu et al. [7], tried to develop a prediction rule as an alternative to microbiological tests to aid clinicians in diagnosing typhoid fever. The sensitivity and specificity of their prediction rule were 83.8% and 82.4%, respectively; however, their prediction rule included the Widal test result and leukocyte count [7]. Compared to other studies, this study showed that the Nelwan Score, a scoring system based on clinical manifestations, had acceptable diagnostic value and better practicality in the absence of laboratory investigations.

We then compared our study with the study conducted by Neopane et al. [8], who also developed diagnostic criteria for typhoid fever based on clinical manifestations. The sensitivity and specificity of their diagnostic criteria were 72.2% and 98.3%, respectively. They pointed out that their diagnostic criteria were unique, as they avoided laboratory investigations. This study, which has a larger sample size, also evaluated a scoring system based on clinical manifestations, and found that the Nelwan Score, had good diagnostic value with better sensitivity (81.8%). This suggests that the Nelwan Score might be a better clinical screening tool for patients with suspected typhoid fever. Furthermore, this score can be extensively implemented as a typhoid fever screening tool in healthcare facilities with limited resources.

Statistically, patients with a total score of 10 were estimated to have a confirmed typhoid fever case with sensitivity, specificity, positive predictive value (PPV), and NPV of 81.8%, 60.8%, 9.3%, and 98.5%, respectively. The positive and negative likelihood ratios of the Nelwan Score in this setting are 2.086 and 0.299, respectively. The PPV was very low due to the low proportion of patients with typhoid fever in this study. However, our findings suggest that when a patient with suspected typhoid fever has consistent clinical signs and symptoms with a Nelwan Score of ≥ 10, clinicians can promptly initiate treatment in setting where laboratory investigations such as culture are not available. In patients in whom the Nelwan Score is < 10, laboratory investigations should be performed to confirm the typhoid diagnosis. Using cut-off values of 11 and 12 showed no statistically significant increase in the sensitivity, specificity, and AUC value, compared with a cut-off value of 10, and therefore we suggest a cut-off value of 10 should be used to diagnose typhoid based on the Nelwan Score.

Our study has some limitations. The proportion of confirmed typhoid fever cases was low (4.72%), similar to a recent study by Gasem et al. [23]. Although this may not be a representative figure of typhoid fever in Indonesia, it is comparable to the proportion reported by Punjabi et al. [24] in a study conducted in North Jakarta hospitals in patients admitted with a history of fever for 3 days or more accompanied by abdominal complaints. To date, few studies have reported the prevalence of typhoid fever. This is probably due to difficulties in confirming the diagnosis, because PCR testing is not recommended as a routine diagnostic procedure.

The low incidence of typhoid fever may also be influenced by antibiotic administration prior to conducting laboratory investigations. Several studies have reported that antibiotic administration and the duration between the onset of the disease and sample collection contributed to the low detection rate of typhoid cases [25, 26]. To prevent this from biasing our results, we restricted the eligibility criteria to patients without current antibiotic use. However, we learned that it is essential to ask for more specific information regarding current antibiotic exposure because patients might have already visited the outpatient clinic before being included in our study.

Another reason why only a small proportion of participants were confirmed with typhoid fever is that culture (especially rectal swab culture) has limited sensitivity, which varies throughout the course of the disease (40–80% during the incubation period) [25, 27]. The same factors also apply to PCR. Although some studies have shown that PCR has high sensitivity and specificity, false-negatives may still occur [28–30]. False-negative PCR results may result from the small numbers of bacteria, gene mutations, and inhibitory substances in the sample [30].

Another limitation of this study is that it did not evaluate the items of the Nelwan Score as a scoring system. Nevertheless, this study showed that the Nelwan Score as an appropriate method in which clinical signs and symptoms may be appropriately recognized to diagnose typhoid fever.

## Conclusion

The clinical features used in the Nelwan Score showed that a cut-off value of 10 was the score with the best diagnostic value. Taking this into consideration, the Nelwan Score is applicable as a screening tool for patients presenting with suspected typhoid fever features in settings where laboratory resources are limited and could reduce the inappropriate use of antibiotics in patients with suspected typhoid.

## Supporting information

S1 Dataset Original dataset. (XLSX)

S1 File STARD flow diagram. (DOCX)
