# Supplementary material for: Validation of the Nelwan Score as a screening tool for the diagnosis of typhoid fever in adults in Indonesia
Source: PLoS One. 2023 May 12;18(5):e0256508. doi: 10.1371/journal.pone.0256508 (PMC10180627; doi:10.1371/journal.pone.0256508)
Supplement: S1 File — (DOCX) [file pone.0256508.s002.docx]

**S2.** **STARD Flow Diagram showing the numbers receiving score examination and reference tests.**

Reference test negative

Non-confirmed case of typhoid fever

(*n* = 222)

Eligible Patients

(Fever and Gastro Intestinal Complaint)

(*n* = 233)

History taking and physical examination

by clinicians using score items (*n* = 233)

**Reference Tests**

by laboratory investigators (*n* = 233)

- Blood culture (*n=*233)
- Blood PCR (*n*=233)
- Rectal swab culture (fever > 1 week) (*n*=31)

Reference test positive

Confirmed case of typhoid fever

(*n* = 11)
